# The therapeutic potential of vitamins as nutrients in food for anti-inflammatory and anti-oxidative stress in liver fibrosis diseases

**DOI:** 10.3389/fnut.2026.1783979

**Published:** 2026-03-27

**Authors:** Wanze Feng, Zeyuan Zhao, Zengxia Liu, Chunlei Hua, Shuqi Zheng, Haibin Wang, Ying Xia, Minhui Li

**Affiliations:** 1 College of Pharmacy, Baotou Medical College, Baotou, China; 2 College of Basic Medicine, Inner Mongolia Medical University, Hohhot, China; 3 Central Laboratory, Inner Mongolia Autonomous Region Hospital of Traditional Chinese Medicine, Hohhot, China

**Keywords:** inflammation, liver fibrosis, medicine food homology, oxidative stress, vitamins

## Abstract

Inflammation and oxidative stress are the key pathogenic mechanisms for the occurrence and development of liver fibrosis. After liver cell damage, excessive reactive oxygen species (ROS) are released, which is a key trigger for the activation of hepatic stellate cells (HSCs). Once activated, HSCs transform into myofibroblasts, leading to excessive extracellular matrix (ECM) deposition and promoting the formation of liver fibrosis. The above processes interact with each other, mutually amplifying and forming a vicious cycle, jointly accelerating the development process of liver fibrosis. The commonly used antioxidants in clinical practice, such as SOD, CAT, and anthocyanins, play a significant role in clinical antioxidant therapy. Vitamins, as common and natural antioxidants, have the characteristic of being both food and medicine, and are relatively safe. Compared with the commonly used antioxidants in clinical settings, vitamins have higher bioavailability, lower adverse reactions and side effects, are widely available, and are easy to obtain. Patients with liver fibrosis often suffer from multiple vitamin deficiencies due to reduced intake, absorption disorders, and increased consumption. This may further accelerate the disease progression. However, the anti-inflammatory and antioxidant effects of vitamins in liver fibrosis and their underlying mechanisms have not been fully elucidated. Moreover, systematic studies on various vitamins are still relatively scarce. Based on the above background, this article systematically elaborates on the research progress of various vitamins in the prevention and treatment of liver fibrosis through their antioxidant and anti-inflammatory mechanisms, with a focus on correcting the deficiency state. The aim is to provide a theoretical basis for precise nutritional intervention for patients with liver fibrosis due to vitamin deficiency.

## 1 Introduction

As a kind of essential micronutrients, vitamins play an important role in human metabolism and disease prevention. As a natural antioxidant, vitamins are widely available and easy to obtain compared with other antioxidants. Antioxidants commonly used in clinical practice include free radical absorbent antioxidants, such as lycopene, lutein, tea polyphenols, etc. Peroxide decomposer antioxidants, such as N-acetylcysteine, peroxide reductase, etc. Enzyme antioxidants, such as SOD and CAT, play an important role in clinical anti-oxidation. As a common and natural antioxidant, vitamin has the characteristics of both food and medicine as a drug and food homologous substance, and has high safety (1, 2). Compared with the antioxidants commonly used in clinical practice, vitamins have high bioavailability, low adverse reactions and side effects.

**Figure 1:**
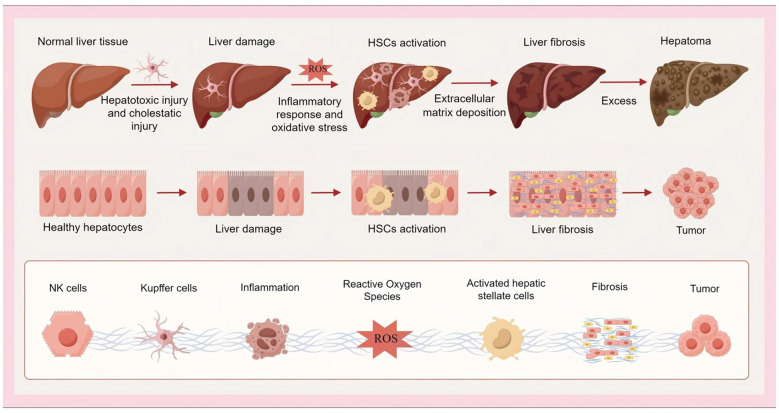
Pathological process of liver fibrosis.

Liver fibrosis refers to the pathological connective tissue hyperplasia in the process of chronic injury repair of the liver, mainly manifested as abnormal deposition and structural remodeling of extracellular matrix (ECM) (3). The onset of liver fibrosis begins with liver toxicity or cholestatic injury. Hepatotoxic injury is usually associated with viral hepatitis, alcohol abuse or non-alcoholic steatohepatitis (NASH), while cholestatic injury is common in cholangitis or biliary atresia (4). Inflammation and oxidative stress induced by injury, and then activation of hepatic stellate cells (HSCs) is the core step. Activated HSCs produce a large number of collagen fibers and inhibit their degradation, resulting in excessive deposition of ECM, and ultimately the formation of liver fibrosis. If this process is not intervened, it can progress to cirrhosis, liver failure and even hepatocellular carcinoma (5–7) (Figure 1). As a common pathological pathway of chronic liver disease, liver fibrosis has become a major public health burden due to the increasing global morbidity and mortality (8).

Inflammation and oxidative stress are the key pathogenic mechanisms of the occurrence and development of liver fibrosis. Excessive reactive oxygen species (ROS) released after hepatocyte injury is a key trigger for HSCs activation. HSCs are converted into myofibroblasts after activation, resulting in excessive deposition of ECM and fibrosis (9). Chronic inflammation caused by persistent liver injury (such as chronic hepatitis or steatohepatitis) will further induce oxidative stress and DNA damage in hepatocytes. The two interact and amplify each other to form a vicious circle and jointly promote the development of liver fibrosis (9, 10).

The vitamin intervention discussed in this article mainly targets a specific group of people, namely patients with liver fibrosis. Patients with chronic liver diseases often fall into a state of vitamin deficiency due to multiple factors such as decreased appetite, insufficient dietary intake, intestinal absorption disorders (such as cholestasis), decreased liver reserve function, and increased oxidative stress consumption. Therefore, vitamin intervention should precisely focus on subgroups of patients with liver fibrosis who have a clear risk of deficiency. The effects of vitamins discussed in this review are mainly based on the perspective of correcting secondary deficiencies and restoring physiological homeostasis, rather than pharmacological interventions aimed at achieving supra-physiological effects in nutrient-sufficient populations. However, the potential functions and mechanisms of vitamins in anti-inflammatory and antioxidant properties in liver fibrosis have not yet been fully elucidated, especially for comprehensive studies on various vitamins. Given this background, this review aims to systematically elaborate on the therapeutic potential of vitamins as dietary nutrients in regulating inflammation and oxidative stress in liver fibrosis diseases, with the aim of providing a theoretical basis for precise nutritional intervention for patients with vitamin-deficient liver fibrosis.

## 2 Inflammation and oxidative stress in liver fibrosis

Oxidative stress is a state of redox imbalance caused by excessive production of ROS, such as superoxide anion ${\text{O}}_{2}^{-}$, hydroxyl radical-OH, and peroxy radical ROO^−^, which has a harmful effect on cell components and promotes programmed cell death (11). Excessive production of ROS activates inflammatory signaling pathways, which in turn promotes the release of inflammatory cells and mediators, leading to the occurrence of chronic inflammation (12). Although inflammatory response is an important host defense mechanism, its excessive activation may trigger widespread immunopathological changes and tissue damage, aimed at responding to exposure of tissues or organs to harmful stimuli (such as trauma, invasion of pathogenic microorganisms), by triggering a cascade of cellular and molecular mechanisms to respond to damage and initiate the process of healing (13, 14). It usually involves the acute phase, including the initiation phase (activation of immune cells and chemical mediators) and the resolution phase (restoration of homeostasis), which is the host 's self-protection mechanism (15). However, if inflammation is not resolved properly, it may be out of control and turn into a chronic state, leading to immunopathological damage and tissue destruction. In the inflammatory state, it will further promote the production of ROS, aggravate tissue oxidative damage, form a vicious circle, and continue to aggravate tissue damage and pathological changes (16, 17). This mutually reinforcing vicious cycle occurs repeatedly in many chronic diseases such as chronic liver disease and diabetic nephropathy, and becomes the core mechanism of disease development (18, 19). Studies have shown that oxidative stress is a key initiator of nonalcoholic fatty liver disease, excessive free fatty acid (FFA) accumulation and mitochondrial dysfunction (20). FFA produces excessive ROS through the β-oxidation process, destroying the oxidation/antioxidant balance, leading to lipid peroxidation, ERS and mitochondrial damage in hepatocytes. This oxidative damage directly causes hepatocyte death and activates related inflammatory signaling pathways (21, 22). Inflammation is triggered by excessive production of ROS, leading to liver cell damage and the release of inflammatory cytokines. This process promotes the transformation of non-alcoholic fatty liver disease from simple steatosis to liver fibrosis. Its pathological changes include sustained inflammatory response after liver cell pathology, recruitment of macrophages and activation of HSCs, resulting in excessive deposition of ECM and triggering fibrosis response (23–25). In diabetes, insulin resistance and hyperglycemia promote ROS accumulation, weaken the antioxidant defense system, induce inflammatory response, and aggravate disease progression (26).

The pathological feature of liver fibrosis is a repair process (such as viral hepatitis, alcohol abuse or metabolic diseases) that occurs when the liver responds to chronic injury. Activation of HSCs and excessive deposition of ECM are important links and constitute the pathological basis for the development of liver cirrhosis and liver cancer (27). In liver fibrosis disease, inflammation is one of the key driving factors. Chronic liver injury (such as chronic hepatitis or non alcoholic steatohepatitis) leads to liver cell death or damage (28), releasing the damage related molecule HMGB1, activating inflammation related signaling pathways, and stimulating monocytes, macrophages, and neutrophils into the liver. These immune cells secrete pro-inflammatory cytokines, which in turn initiate an inflammatory cascade reaction (29). HSCs are vitamin A storage cells in a quiescent state in the normal liver. Inflammatory factors activate nuclear NF-κB and mitogen-activated protein kinase (MAPK) signaling pathways by binding to receptors on the surface of HSCs, leading to the transformation of HSCs into activated myofibroblast-like cells (30, 31). These activated HSCs begin to proliferate, leading to a large deposition of ECM and secretion of more pro-inflammatory factors, forming a positive feedback loop and exacerbating liver fibrosis (32). Studies have found that NF-κB signaling pathway plays a central role in mediating the activation of inflammatory HSCs, and inhibition of NF-κB can significantly reduce the progression of inflammation and fibrosis (33). ROS is the main effector molecule of oxidative stress, produced by a variety of sources. In liver fibrosis, ROS is mainly produced by damaged liver cell mitochondria, activated immune cells, and HSCs. The key enzymes include nicotinamide adenine dinucleotide phosphate hydrogen oxidase 4 (NOX4), which is the main source of ROS in liver fibrosis and upregulated in chronic liver injury (34, 35). When the liver is damaged and mitochondrial electrons leak, NADPH oxidase produces superoxide anion (O^2−^), and ROS enters the cytoplasm. Mn-SOD in mitochondria and Cu/Zn SOD in cytoplasm convert superoxide radicals into H_2_O_2_ and O_2_, protecting mitochondrial structure (36). Glutathione forms a cycle in the cytoplasm and mitochondria through glutathione peroxidase and glutathione reductase (37). Cell membrane vitamin E captures LOO^−^, vitamin C reduces vitamin E, self oxidizes to dehydroascorbic acid (DHA), and is then reduced to vitamin C. In the resting state, Nrf2 binds to Keap1, ROS causes Nrf2 to enter the nucleus, and antioxidant enzymes are expressed (38). In addition, mitochondrial dysfunction and endoplasmic reticulum stress (ERS) are important sources of ROS, which can directly oxidize lipids, cell membranes, endoplasmic reticulum, mitochondria and DNA, leading to liver cell damage and death (39). Increased ROS levels activate HSCs by enhancing mitochondrial activity and up-regulating NOX. Activated HSCs further activate the MAPK/ERK pathway and SMAD transcription factors, and aggravate the redox imbalance through NOX enzymes, ultimately promoting collagen production and accelerating the process of liver fibrosis (40).

**Figure 2:**
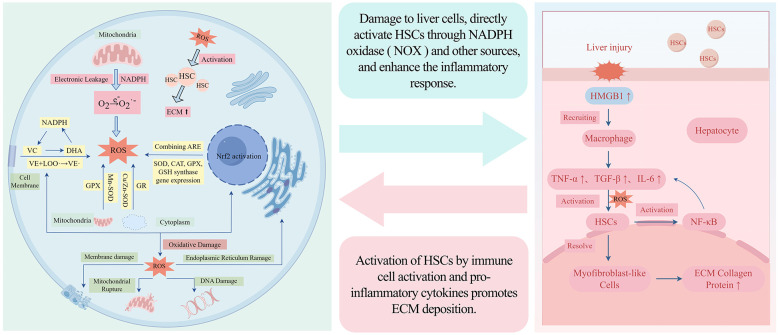
Nausea cycle of inflammation and oxidative stress in liver fibrosis disease.

The key pathogenic mechanism of the occurrence and development of liver fibrosis is the interaction between inflammatory response and oxidative stress. Inflammation activates HSCs through immune cell activation and pro-inflammatory cytokines, promoting ECM deposition; ROS generated by oxidative stress can damage liver cells, directly activate HSCs through sources such as NADPH oxidase (NOX), and enhance inflammatory response. The two are interrelated and amplify each other, forming a synergistic vicious cycle that jointly promotes the occurrence and development of liver fibrosis disease (Figure 2).

## 3 Introduction to vitamins

As an essential micronutrient for human body, vitamins play an important role in the metabolic process and disease prevention and treatment. As a natural antioxidant, vitamins are widely available and easy to obtain compared with other antioxidants. In terms of oral absorption and utilization of vitamins, the absorption and utilization rate is high and the bioavailability is good. In contrast, other antioxidant molecules are larger, the absorption and utilization is poor, and the bioavailability is relatively low. The side effects of vitamins are also relatively small and safe at normal doses, while other antioxidants may have large side effects at therapeutic doses, limiting their long-term use (41–43). The cost of vitamin supplementation through diet is much lower than that of artificial preparations, which highlights unique advantages in reducing medical burden and improving patient compliance. These characteristics together indicate that the model of maintaining continuous physiological concentration through food intake of vitamins is more in line with the regulatory needs of chronic inflammation and oxidative stress than the drug intervention model. This makes vitamins have unique advantages in the field of antioxidant therapy, which is worthy of further research and application.

Vitamins can be divided into fat-soluble and water-soluble according to their solubility. The former includes vitamin A, D, E and K, and the latter includes vitamin B group and vitamin C. Each vitamin plays an irreplaceable physiological function in the body. Fat-soluble vitamins are a class of vitamins that are insoluble in water but soluble in fats and organic solvents. They have a long half-life, so they can maintain effective concentrations in the body and continue to play a role without frequent intake. It has a wide range of natural dietary sources, including fruits, vegetables, nuts and animal foods. Unlike fat-soluble vitamins, water-soluble vitamins are soluble in water, have a short half-life and cannot be stored in the body for a long time. Their good sources include cereals, vegetables, fruits, meat, beans and other foods.

### 3.1 Mechanisms of anti-inflammatory and antioxidant effects of fat-soluble vitamins in liver fibrosis diseases

Fat-soluble vitamins all contain hydrophobic groups in their chemical structures and need to be taken together with dietary fats to promote absorption. They have a relatively long half-life in the body and are mainly metabolized and stored by the liver. These common pharmacokinetic characteristics make them more prone to deficiency in patients with liver fibrosis, especially those with cholestasis or absorption disorders. At the molecular mechanism level, fat-soluble vitamins regulate the activation of HSCs, ECM metabolism, and transcription of genes related to inflammation and oxidative stress through receptors such as RAR/RXR, VDR, and PXR, as well as signaling pathways such as NF-κB and AMPK. However, there are significant differences in the specific mechanisms of action of fat-soluble vitamins. The following are the specific mechanisms for each type of fat-soluble vitamin.

#### 3.1.1 Vitamin A

Vitamin A mainly includes retinol, retinol and retinoic acid, which are mainly present in animal foods and can be directly used by the human body. Vitamin A plays an important role in maintaining human physiological homeostasis, including visual acuity maintenance, immune regulation and cell differentiation, and is closely related to liver fibrosis (44). In the liver, vitamin A is mainly enriched in the lipid droplets of HSCs in the form of retinyl ester. Therefore, HSCs are regarded as the storage cells of vitamin A, which helps to maintain the normal function and metabolic balance of the liver (45).

The liver is the core place for vitamin A metabolism and storage, and HSCs are regarded as professional storage cells for vitamin A in the liver. Retinoic acid is the main storage form of vitamin A in lipid droplets, which helps to maintain the normal function and metabolic balance of the liver (46). When the liver is damaged, HSCs are activated, changing from a static vitamin A-rich state to a myofibroblast-like phenotype. In this process, vitamin A storage is significantly degraded and lost (47). Activated HSCs lose their unique lipid droplet characteristics and promote the accumulation and fibrosis of extracellular matrix proteins (48). Vitamin A and its metabolites and related receptors play a key regulatory role in HSCs activation. RXRα is a key regulator of HSCs activation and liver fibrosis. RXRα regulates the activation of AMPKα mediated by Ca MKKβ (49). Hematopoietic stem cells are responsible for the effector cells of liver fibrosis, in which part of vitamin A is stored in the form of retinol (50). Hematopoietic stem cells differentiate into activated myofibroblasts, which is associated with the storage and loss of retinoids in hematopoietic stem cells (51). Through the retinol-like signal transduction of RAR and RXR, the interference of hematopoietic stem cells may lead to the activation of fibrogenesis of hematopoietic stem cells (52). In addition, retinoic acid can regulate the expression of pro-fibrotic markers and affect the differentiation of inflammatory cells, thereby regulating the process of fibrosis and inflammation. When the level of vitamin A is sufficient, it can inhibit the pro-inflammatory signaling pathway JAK/STAT, and inhibit the aggregation of inflammatory cells such as macrophages, thereby reducing the inflammatory load of liver fibrosis (53). Retinoic acid has a significant down-regulation effect on oxidative stress markers in lipopolysaccharide-induced liver injury experiments, such as by regulating iron metabolism related proteins to reduce ROS production, thereby protecting liver tissue from oxidative damage (54). Studies have shown that retinoic acid can regulate MAPK, AMPK and other signaling pathways, which are closely related to the maintenance of cell redox state. The protective effect of vitamin A on the liver is more likely to be due to its combined effects of multiple mechanisms such as maintaining the resting state of HSCs and inhibiting pro-inflammatory signaling pathways (such as JAK/STAT). Clinical studies have shown that patients with liver fibrosis and cirrhosis are often accompanied by a decrease in vitamin A levels, especially in alcoholic and non-alcoholic steatohepatitis (NASH) -related cirrhosis (55). All-trans retinoic acid (atRA), as an active metabolite of vitamin A, shows anti-fibrotic properties in animal models and *in vitro* experiments. It can inhibit the transformation of hepatic stellate cells (HSCs) into myofibroblasts by regulating the activation of HSCs, thereby reducing the excessive deposition of extracellular matrix and alleviating liver fibrosis (56). High dietary vitamin A intake can also reduce the risk of liver cancer (57).

Vitamin A regulates both inflammation and oxidative stress in liver fibrosis through its active metabolites such as retinoic acid. It reduces inflammatory responses by inhibiting pro-inflammatory cytokines and the differentiation of inflammatory cells, and enhances antioxidant defense by maintaining cellular redox homeostasis. Thus, supplementing vitamin A levels in patients with liver fibrosis can delay the progression of the disease, while its deficiency will increase the risk of liver fibrosis and related diseases.

#### 3.1.2 Vitamin D

Vitamin D is an essential nutrient for the human body. It mainly includes vitamin D2 (ergocalciferol) and vitamin D3 (cholecalciferol). Vitamin D2 is mainly synthesized by plants under ultraviolet irradiation. Humans can supplement vitamin D2 by eating foods such as mushrooms (58, 59). Vitamin D3 in the human body is mainly derived from the conversion of 7-dehydrocholesterol in the skin after ultraviolet irradiation (60). In the liver, vitamin D is metabolized to (25 (OH) D) by vitamin D25-hydroxylase (CYP2R1 and CYP27A1), which is the main circulating form of vitamin D (61). (25 (OH) D) is mainly stored in the renal proximal tubules, and 1 α, 25 dihydroxyvitamin D hydroxylase (1 α, 25 (OH) D-hydroxylase) is metabolized into 1- α, 25 dihydroxyvitamin D (1 α, 25 (OH) 2D). Finally, (1α, 25 (OH) 2D) was metabolized into water-soluble inactive substances under the action of CYP24A1, and finally excreted through bile and kidney (62).

Vitamin D mainly exerts multi-level anti-fibrotic effects in the liver through its nuclear receptor, the Vitamin D receptor (VDR). In the central effect cells of liver fibrosis, HSCs, after binding with active vitamin D (1, 25(OH)_2_ D_3_) and VDR, form an heterodimer with retinoic X receptor (RXR). This complex can directly antagonize the fibrotic TGF-β/Smad signaling pathway, competitively inhibit the binding of Smad3 to DNA, thereby directly blocking the activation of HSCs and their transformation into myofibroblasts at the transcriptional level, and reducing the generation of ECM such as type I collagen (63–65). In addition to direct inhibition of HSCs, the vitamin D-VDR signaling pathway also indirectly hinders the progression of fibrosis by reshaping the liver immune microenvironment and maintaining metabolic homeostasis. On one hand, VDR activation can regulate the functions of liver Kupffer cells, inhibit the release of pro-inflammatory cytokines (such as TNF-α and IL-6) mediated by NF-κB, and alleviate the continuous activation of HSCs driven by chronic inflammation (66–68). On the other hand, vitamin D upregulates the expression of TXNIP in bile duct cells and HSCs, inhibits the generation of ROS mediated by the thioredoxin system, thereby reducing oxidative stress to liver cells and their paracrine activation of HSCs (69, 70). Maintaining a normal and high level of vitamin D not only improves liver insulin resistance, but also reduces the pro-inflammatory and pro-fibrotic responses in the liver and adipose tissues (71). Supplementation with vitamin D can also improve the mitochondrial abnormalities in senile fatty liver, reduce MDA accumulation, maintain the activity of antioxidant enzyme SOD, and thereby block the fibrotic process driven by oxidative stress (72, 73). Clinical studies have also confirmed that patients with chronic liver diseases generally suffer from vitamin D deficiency. Supplementing with vitamin D can increase the level of serum 25(OH)D, which helps to slow down the progression of liver fibrosis and cirrhosis (74–77). Vitamin D can also exert anti-cancer effects by inhibiting the proliferation of tumor cells and inducing apoptosis (78). Therefore, maintaining adequate levels of vitamin D, especially in patients with liver fibrosis, may be an effective adjunctive treatment strategy.

In conclusion, vitamin D, through its synergistic effects of directly inhibiting the activation of HSCs, anti-inflammatory, antioxidant, and metabolic regulation, can effectively break the vicious cycle of inflammation, oxidative stress, and HSC activation in the process of liver fibrosis.

#### 3.1.3 Vitamin E

The vitamin E family consists of four tocopherols (αT, βT, γT, δT) and four tocotrienols (αTE, βTE, γTE, δTE). Among them, α-tocopherol (αT), as the main form of vitamin E in tissues, is essential for maintaining neurological function. Its deficiency can lead to ataxia (79). α-tocopherol is recognized as the most important active form, with complete vitamin activity, while other forms are less active. The structure of vitamin E includes tryptamine group, which endows it with antioxidant properties (80). Vitamin E is mainly found in vegetable oils, nuts and seeds. Dietary sources are the main way for the human body to obtain vitamin E (81). The liver is the metabolic center of vitamin E, which is responsible for secreting α-tocopherol into plasma for lipoprotein transport, and preferentially metabolizes other forms of vitamin E (82, 83). Vitamin E is mainly absorbed in the intestine (84), and then the metabolic pathway is mainly dominated by ω-hydroxylase-mediated side chain oxidation, forming 13 ' -hydroxychromanols and long-chain metabolites (LCMs), which are finally excreted with urine and feces (85, 86).

Vitamin E, as a natural antioxidant, can inhibit hematopoietic stem cells to alleviate liver fibrosis (87). Vitamin E is mainly stored and metabolized in the liver, and it has a regulatory relationship with hematopoietic stem cells. Through the regulation of gene expression, dietary vitamin E can down-regulate the expression levels of c-myc gene and TGF-α, thereby reducing the content of iNOS and NADPH oxidase, which is the key cause of oxidative stress (88, 89). In addition, vitamin E can inhibit peroxidation and inhibit HSCs activation through its antioxidant effect, thereby reducing the expression of TGF-β, which is associated with liver fibrosis and hepatocyte apoptosis (90). Vitamin E can directly affect the production of inflammatory factors and the activation of inflammatory pathways through molecular mechanisms such as lipid metabolism and immune regulation.α-tocopherol can reduce inflammation in the liver by inhibiting JNK/c-Jun, and inhibit JNK/CHOP apoptosis signal to reduce hepatocyte apoptosis, while down-regulating autophagy overactivation and HSCs activation, thereby reducing liver fibrosis (91). Vitamin E supplementation can increase adiponectin gene and protein expression (92). Adiponectin can improve nonalcoholic fatty liver disease by inhibiting liver fatty acid synthesis and reducing inflammation, thereby preventing the further development of liver fibrosis into cirrhosis and even liver cancer (93). The gut microbiota-biliary acid axis has been shown to be a key pathway for the progression of liver fibrosis. Biliary drainage disorder can cause intestinal flora imbalance, destroy the intestinal mucosal barrier, promote bacterial translocation and activate the immune response, and accelerate the process of fibrosis (94). As an important metabolite of intestinal flora modification, bile acid is involved in the regulation of intrahepatic inflammation and fibrosis through signal pathways such as farnesoid X receptor (FXR) (95, 96). Previous studies have shown that alpha-tocopheryl quinone treatment significantly impaired liver damage and fibrosis in CCl4-treated mice, with a noticeable increase in the gut microbiota. Mechanistically, alpha-tocopheryl quinone activated the intestinal FXR/FGF15 pathway. Abx and FMT experiments confirmed the microbiota-dependent antifibrotic effects of alpha-tocopheryl quinone. Focusing on the critical role of intestinal FXR signaling (97). A number of randomized controlled trials (RCTs) and meta-analysis have shown that vitamin E, whether used alone or in combination, can significantly reduce serum ALT and AST levels, thereby reducing liver injury (98). Although the clinical evidence of vitamin E in the treatment of cirrhosis is insufficient, vitamin E supplementation can delay disease progression in patients with non-alcoholic fatty liver disease with bridging fibrosis or early cirrhosis (99).

In summary, vitamin E can not only inhibit the activation of hematopoietic stem cells and HSCs through its strong antioxidant effect, thus resisting liver fibrosis, but also play an anti-fibrosis role in multiple aspects by regulating lipid metabolism and affecting intestinal flora. In the future, it is necessary to further explore whether α-tocopherol plays an anti-fibrosis role by reshaping the structure of intestinal flora and regulating bile acid metabolism, so as to provide new ideas for precise intervention of liver fibrosis. Therefore, for patients with liver fibrosis, supplementing with vitamin E can inhibit the activation of hematopoietic stem cells and HSCs through its antioxidant effect, reduce inflammatory responses, and exert anti-fibrotic effects.

#### 3.1.4 Vitamin K

Vitamin K mainly includes vitamin K1 (chlorophyll quinone) and vitamin K2 (menaquinone). The sources of vitamin K1 and K2 are different. The former mainly comes from green leafy vegetables and vegetable oil, while the latter is synthesized by intestinal flora and is found in fermented and animal foods (100). Vitamin K plays an important physiological function in the human body. The most important role is to participate in the blood coagulation process and prevent bleeding as an essential coenzyme for the synthesis of coagulation factors (101). Vitamin K is mainly absorbed in the intestine and transported to different tissues through lipoproteins (102). Intramuscular injection of vitamin K1 can effectively prevent neonatal vitamin K deficiency hemorrhage, and high-dose vitamin K can reverse the anticoagulant effect caused by warfarin overdose (103, 104).

The liver plays a central role in the process of human coagulation. Acute and chronic liver diseases are highly correlated with coagulation disorders (105). Vitamin K is involved in the process of blood coagulation, which can alleviate the coagulation effect of patients with abnormal liver function (106). The core physiological function of vitamin K is to act as a cofactor of γ-glutamyl carboxylase, catalyzing the carboxylation modification of specific glutamic acid residues, which is essential for the activation of coagulation factors (such as prothrombin) and some bone metabolic proteins. In recent years, studies have explored the potential role of vitamin K in the regulation of inflammation. Preliminary evidence shows that vitamin K can activate Pregnane X receptor (PXR). PXR was originally considered to be the main regulator of hepatic xenobiotic metabolism, and has become a key regulator of intestinal homeostasis, inflammation, and oxidative stress. Vitamin K shows therapeutic potential in promoting liver detoxification, strengthening intestinal barrier and regulating pro-inflammatory and apoptotic pathways by activating PXR receptors (107). This suggests that vitamin K may inhibit liver inflammation through the PXR signaling pathway and indirectly alleviate the process of liver fibrosis driven by inflammation. Vitamin K (MK-4) can inhibit the expression of NOS-2, COX-2, MMPs and pro-inflammatory cytokines (TNF-α, IL-1β, IL-6, IL-8) in mouse macrophages. The short-chain isoprene side chain form shows stronger anti-inflammatory potential (108). In the DSS-induced mouse colitis model, vitamin K2 significantly down-regulates IL-1β, IL-6, and TNF-α mRNA, up-regulates the anti-inflammatory factor IL-10, and maintains intestinal barrier integrity (109). Some studies have indicated that in the rat aging model, vitamin K2 can significantly reduce the expression of inflammatory factors such as TNF-α, COX-2, and iNOS in liver tissue by regulating the Keap-1/Nrf-2/HO-1 signaling pathway, while alleviating liver cell apoptosis and fibrosis, improving liver function and mitochondrial ultrastructure, suggesting that vitamin K2 has potential to alleviate liver inflammation (110). The carboxylation-independent function of vitamin K also includes participating in ATP production as an electron carrier and preventing ferroptosis (111), mainly from cell-level studies, and its significance in liver fibrosis remains to be verified. Ferroptosis has been shown to be involved in the progression of liver fibrosis (112, 113), but whether vitamin K exerts liver protection by inhibiting ferroptosis remains to be confirmed by further clinical studies. Therefore, current evidence on the anti-inflammatory or antioxidant effects of vitamin K in liver fibrosis should be considered preliminary and exploratory. In clinical studies, vitamin K2 reduces hepatic steatosis and lipid metabolism disorders by regulating liver lipid metabolism, inhibiting fat accumulation and inflammatory response (114). Vitamin K deficiency is caused by cholestasis or intestinal absorption disorder in patients with cirrhosis. Intravenous injection of vitamin K1 can significantly reduce the coagulation function index INR (115). Prophylactic use of vitamin K1 can also reduce the risk of upper gastrointestinal rebleeding (116).

Therefore, for patients with liver fibrosis, supplementing with vitamin K can protect liver cells by activating the PXR receptor and through mechanisms such as antioxidative stress-mediated ferroptosis, thereby alleviating liver fibrosis.

### 3.2 Anti inflammatory and antioxidant mechanisms of water-soluble vitamins in liver fibrosis diseases

Unlike fat-soluble vitamins, which exert long-term transcriptional regulation through nuclear receptors, water-soluble vitamins (C and B group) are mainly used as coenzymes or cofactors to participate in the immediate regulation of energy metabolism, one-carbon transfer and redox balance. Its role in liver fibrosis is more reflected in the maintenance of basal metabolic homeostasis, which indirectly affects inflammation and oxidative stress.

#### 3.2.1 Vitamin C

Vitamin C, also known as ascorbic acid, is found in fresh fruits and vegetables. Vitamin C not only has an antioxidant effect, but also has a key role in regulating lipid metabolism, promoting angiogenesis, and promoting collagen synthesis to stabilize the extracellular matrix (117). Vitamin C enters the intestine through the stomach after ingestion through food, and enters the blood circulation through the liver through the sodium-dependent transporter (SVCT1/2), which is distributed throughout the body tissue (118). Vitamin C plays a key role in antioxidant defense. Its strong reducing properties maintain redox homeostasis by neutralizing free radicals, regenerating vitamin E, and participating in enzymatic reactions (119, 120).

Vitamin C plays an antioxidant role by providing electrons to directly neutralize ROS and free radicals. The enediol structure in its molecule makes it a strong electron donor, which is converted into DHA during oxidation, thereby protecting biomolecules and tissues from oxidative stress damage (121). Normalization of the quality of hematopoietic stem cells in the liver is an important strategy for the treatment of liver fibrosis (122), and TGF-β1 is essential for the development and division of hematopoietic stem cells. Therefore, inhibition of TGF-β1 signaling in hematopoietic stem cells may effectively reverse liver fibrosis (123, 124). The anti-proliferative effect of vitamin C on HSC-T6 is related to the regulation of intracellular TGF-β1 signaling pathway (125). Therefore, *in vitro* experiments, vitamin C may be a potential candidate drug for the treatment of liver fibrosis. In nonalcoholic fatty liver disease (NAFLD) models, vitamin C deficiency is often accompanied by the progression of NAFLD, which may aggravate oxidative stress in the liver (126). As an antioxidant, vitamin C can directly neutralize ROS, reduce liver oxidative damage, and indirectly promote HSCs activation and liver fibrosis disease progression (127). Perfluorooctanoic acid (PFOA) has a damaging effect on the liver, and vitamin C supplementation can reduce PFOA-induced total cholesterol and triglyceride levels and reduce abnormal enlargement of the liver, mainly by inhibiting linoleic acid metabolism and increasing glutathione in the liver (128). Studies have shown that SMP30 knockout mice induce vitamin C deficiency model, and vitamin C supplementation can significantly reduce alcoholic liver injury by inhibiting neutrophil and CD68+ cell infiltration. Vitamin C can be used as a potential anti-inflammatory therapeutic agent for alcoholic liver disease by inhibiting neutrophil infiltration, which helps to reduce alcoholic liver disease and prevent the further transformation of alcoholic liver disease into liver fibrosis (129). When vitamin C is insufficient in the body, liver tissue shows increased deposition of collagen fibers and increased number of activated HSCs. In LX-2 cells and rat primary HSCs, vitamin C can directly inhibit cell proliferation and hydrogen peroxide-induced collagen expression. Vitamin C deficiency aggravates the hepatotoxicity of TAA, which is mainly due to insufficient liver ROS clearance and directly acts on HSCs to promote the progression of liver fibrosis (130). Vitamin C deficiency is more common in patients with upper gastrointestinal bleeding (UGIB), and its deficiency can cause higher mortality, rebleeding and hospital stay. It is necessary to supplement vitamin C in advance to prevent the clinical effects of patients due to lack of vitamin C (131). Clinical studies have shown that there is a non-linear correlation between serum vitamin C levels and the risk of non-alcoholic fatty liver disease. When vitamin C is lower than 0.92 mg/dL, serum vitamin C levels are negatively correlated with the risk of non-alcoholic fatty liver disease (132).

Thanks to its antioxidant properties, for patients with liver fibrosis, supplementing with vitamin C can reduce ROS, enhance the activity of antioxidant enzymes, and inhibit inflammation through multiple mechanisms, thereby alleviating liver oxidative damage and slowing down the progression of liver fibrosis.

#### 3.2.2 Vitamin B group

Vitamin B group mainly includes vitamin B1 (thiamine), vitamin B5 (pantothenic acid), vitamin B6 (pyridoxine), vitamin B7 (biotin), vitamin B9 (folic acid), vitamin B12 (hydrocobalamin), etc., which is an essential nutrient for human body. Vitamin B group is involved in a variety of metabolic processes in the human body, such as carbohydrate, sugar and amino acid metabolism (133, 134).

Vitamin B12 is an essential coenzyme of MTR, which catalyzes the remethylation of Hcy to methionine (135). Vitamin B12 deficiency leads to decreased MTR activity and Hcy accumulation in the body. Hyperhomocysteinemia (HHcy) not only activates the NF-κB pathway and induces the expression of pro-inflammatory cytokines (such as TNF-α, IL-6), but also spontaneously oxidizes itself to produce reactive oxygen species such as superoxide anion and hydrogen peroxide (136). By interfering with the metabolism of methionine and cysteine (the precursor of GSH synthesis), HHcy reduces intracellular GSH levels and weakens antioxidant capacity (137). In liver microcirculation, HHcy damages endothelial cells, promotes local inflammatory cell infiltration, and aggravates inflammation and oxidative damage in liver fibrosis and other lesions (138). Studies have shown that dietary supplementation with vitamin B12 and folic acid can prevent or treat NASH and fibrosis by blocking syntaxin 17 Hcy. This not only restores autophagy but also alleviates the pathological process of NASH (139). Hcy can induce endoplasmic reticulum oxidative stress and activate the unfolded protein response, with downstream signals such as CHOP triggering inflammation and cell apoptosis (140). Therefore, the anti-inflammatory and antioxidant effects of vitamin B12 are indirectly achieved by maintaining normal Hcy metabolism and methylation cycle. SAMe is the most important methyl donor in the body and participates in the methylation modification of DNA, RNA, proteins (including histones), phospholipids, neurotransmitters, etc. SAMe-mediated DNA and histone methylation is an important epigenetic pathway that regulates the expression of inflammatory genes (141). SAMe can participate in the synthesis of phosphatidylcholine, maintaining the membrane integrity and fluidity of HSCs and gastric mucosal epithelial cells, and reducing the inflammatory response caused by cell membrane damage (142). The relationship between vitamin B12 and liver fibrosis is unique, and its level is positively correlated with the degree of fibrosis. Although the level of vitamin B12 may be reduced in advanced patients, higher vitamin B12 levels indicate a higher risk of cirrhosis transformation (143), which brings new challenges to vitamin B12 alone. Therefore, it is recommended to incorporate vitamin B12 into the combination regimen to enhance the efficacy of anti-hepatic fibrosis.

Vitamin B9, also known as folic acid, includes naturally occurring folic acid (found mainly in vegetables, legumes, and animal livers) and the fully oxidized monoglutamate synthetic form, which is widely used in dietary supplements and food fortifiers (144). Vitamin B9 is an essential micronutrient for human body. As a coenzyme of one-carbon transfer reaction, it plays a central role in cell metabolism and participates in DNA synthesis, methylation process and synthesis of nucleic acids, amino acids and SAMe. These processes are essential for cell survival, proliferation and neural development (145–147). The lack of folic acid in pregnant women can lead to fetal malformations. When folic acid is ingested into the human body, it needs to be hydrolyzed into monoglutamic acid by intestinal γ-glutamyl hydrolase and then absorbed by small intestinal epithelial cells. After entering the blood circulation, it enters the cells through RFC and PCFT, and is reduced to DHF and THF under the action of dihydrofolate reductase (DHFR) (148, 149). THF as a carrier participates in the single-carbon metabolic cycle, and forms 5,10-methylenetetrahydrofolate through serine hydroxymethyltransferase (SHMT), which participates in methionine cycle and histidine metabolism (150). As a key organ for folic acid storage and lipid synthesis, the liver has abnormal protein regulation and gene expression when folic acid is deficient. This disorder can lead to triglyceride accumulation and high-density lipoprotein reduction, thus becoming a risk factor for liver disease (151). In the process of hepatitis C, serum folic acid levels gradually decreased with the evolution of the disease from chronic hepatitis, liver fibrosis to liver cancer (152). Folic acid deficiency can induce liver inflammation, lipid metabolism disorders and fat accumulation, thereby promoting fibrosis (153, 154). Folic acid is also a coenzyme. Its core function is to act as a carrier of one-carbon units, participating in the synthesis of purines and pyrimidines, the methionine cycle (homocysteine re-methylation), and methylation reactions. Although some *in vitro* studies have reported that the folic acid molecule may have a certain free radical scavenging ability (155), its core biochemical function at physiological concentrations is as a coenzyme for one-carbon unit metabolism. Therefore, the antioxidant and anti-inflammatory properties of folic acid in the liver mainly result from its indirect metabolic regulatory effect, that is, by maintaining the normal metabolism of homocysteine (Hcy), ensuring the synthesis of S-adenosylmethionine (SAMe), and maintaining the stability of DNA methylation, thereby correcting metabolic disorders, maintaining the stability of gene expression, and ensuring the supply of the precursor for glutathione (GSH), ultimately reducing oxidative damage and inflammatory responses in the liver (156–158).

Vitamin B6 is a key cofactor for glutathione synthesis. In the model of alcoholic liver disease, intestinal flora imbalance will reduce the synthesis of vitamin B6, which in turn interferes with the amino acid metabolism and glutathione synthesis of the liver, and aggravates liver damage (159). Glutathione, as a core antioxidant molecule, its deficiency will aggravate liver oxidative stress and liver fibrosis. The concentration of aspartate transaminase (AST) in serum is commonly used to detect liver cell injury. However, patients with liver fibrosis sometimes experience a decrease in serum AST levels. AST requires active vitamin B6 (PLP) as a coenzyme to express its activity. Supplementing these patients with vitamin B6 may lead to an increase in serum AST concentration, thereby reducing liver fibrosis (160). Patients with cirrhosis and liver fibrosis are often accompanied by vitamin B6 deficiency. Therefore, the level of vitamin B6 in the body is negatively correlated with the degree of liver fibrosis. Vitamin B6 supplementation can slow down the progression of liver fibrosis (161).

Clinical studies have shown that patients with liver injury can lead to vitamin B1 absorption disorders, resulting in delirium and Wernicke encephalopathy. These diseases can be prevented or cured by appropriate vitamin B1 supplements (162, 163). Vitamin B is involved in glucose metabolism and fatty acid oxidation, and its deficiency or insufficient intake may aggravate the progression of liver disease (164).

**Figure 3:**
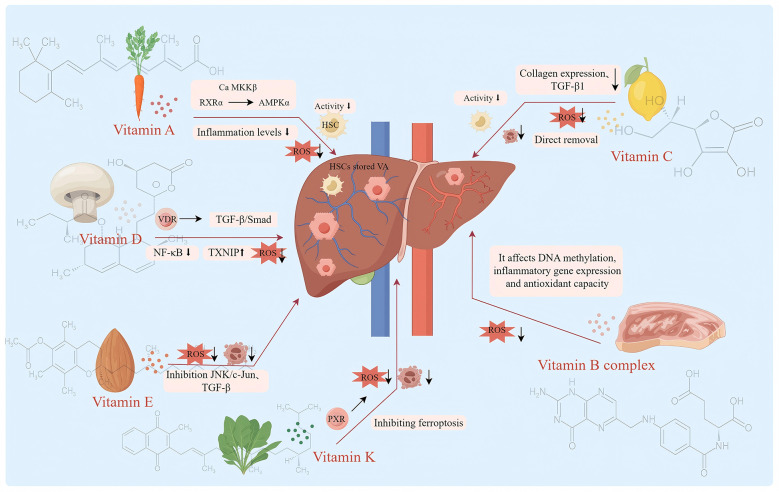
Mechanism diagram of the anti liver fibrosis effect of dietary vitamins (Vitamin A and its metabolites and RXRα exert anti-hepatic fibrosis effects by regulating Ca MKKβ-mediated activation of AMPKα, manifested as inflammatory response and decreased ROS, and inhibition of HSCs activation. Vitamin D antagonizes the TGF-β/Smad signaling through the VDR-RXR complex, inhibits the activation of HSCs and the generation of ECM, suppresses the NF-κB pathway in macrophages, reduces the release of pro-inflammatory factors, upregulates the expression of TXNIP to eliminate ROS, and thereby improves the progression of liver fibrosis diseases. Vitamin E mainly exerts its anti-fibrotic effect by inhibiting the activation of hematopoietic stem cells and HSCs through its antioxidant properties, suppressing inflammatory signaling pathways such as JNK/c-Jun, and reducing inflammatory responses. Vitamin K inhibits liver inflammation and apoptosis by activating PXR receptors, and indirectly inhibits liver fibrosis by inhibiting ferroptosis. Vitamin C can directly remove ROS, regulate inflammatory signaling pathways such as TGF-β1 signaling, inhibit the activation of HSCs and the expression of collagen, thereby inhibiting liver fibrosis. Vitamin B12 and folic acid participate in the prevention and treatment of liver fibrosis indirectly through their key coenzyme roles in the one-carbon metabolism. They maintain the normal conversion of homocysteine (Hcy), ensure the methylation reactions mediated by S-adenosylmethionine (SAMe), and promote the synthesis of glutathione (GSH), thereby stabilizing cellular functions at the metabolic level, indirectly improving the redox balance of cells and inhibiting the formation of inflammatory microenvironments.

In summary, vitamin B groups (especially folic acid and B12) play a role mainly by participating in basic biochemical processes such as one-carbon unit metabolism and amino acid metabolism as coenzymes. Therefore, their antioxidant or anti-inflammatory properties are more likely to indirectly improve the redox state and inflammatory microenvironment of cells by correcting metabolic disorders (such as reducing homocysteine), maintaining genomic stability, and ensuring the synthesis of methylated donor (SAMe) to improve liver fibrosis. Vitamin B6 deficiency can lead to reduced glutathione synthesis, affecting AST activity and other factors, thereby promoting the progression of liver fibrosis (Figure 3).

## 4 Discussion and prospect

Liver fibrosis refers to the phenomenon of pathological connective tissue hyperplasia in the process of chronic injury repair of liver, which is mainly manifested by abnormal deposition and structural remodeling of ECM. When the liver is damaged, inflammation and oxidative stress are produced, HSCs are activated, a large number of collagen fibers are produced, and fiber degradation is inhibited, resulting in excessive deposition of ECM and liver fibrosis. Inflammation and oxidative stress play a key role in the process of liver fibrosis, which not only damages the liver, but also is serious and even life-threatening, greatly reducing the quality of life of patients. Traditional antioxidants have certain limitations in the treatment of inflammation and oxidative stress-related diseases. As a nutrient in food, vitamins have the characteristics of wide sources, high bioavailability, and good patient compliance, and play a key role in maintaining normal physiological functions, regulating metabolic processes, and inhibiting inflammation and oxidative stress.

This article systematically reviews the core mechanisms of various vitamins as natural antioxidants in anti-inflammatory and anti-oxidative stress in liver fibrosis diseases. Vitamin A and its metabolites and RXRα exert anti-hepatic fibrosis effects by regulating Ca MKKβ-mediated AMPKα activation. RAR and RXR can regulate the expression of pro-fibrotic markers, affect the differentiation of inflammatory cells, and control fibrosis and inflammatory processes. The activation of HSCs leads to the degradation of vitamin A storage, which is both a biomarker and part of the disease mechanism, such as the targeted activation of HSCs through vitamin A derivatives to promote resting and reverse fibrosis. Vitamin D antagonizes the TGF-β/Smad signaling through the VDR-RXR complex, inhibits the activation of HSCs and the generation of ECM, suppresses the NF-κB pathway in macrophages, reduces the release of pro-inflammatory factors, upregulates the expression of TXNIP to eliminate ROS, and thereby improves the progression of liver fibrosis. Vitamin E mainly inhibits the activation of hematopoietic stem cells and HSCs through its powerful antioxidant effect, thereby directly combating liver fibrosis. It can effectively alleviate oxidative stress by enhancing the activity of antioxidant enzymes such as SOD. At the same time, it can inhibit key inflammatory and apoptotic signaling pathways such as JNK/c-Jun, and regulate the expression of fibrotic factors such as TGF-β. It can also exert anti-fibrotic effects through regulating lipid metabolism and influencing the composition of the intestinal flora, etc., in multiple aspects. Further research is needed to explore whether α-tocopherol exerts anti-fibrotic effects by reshaping the structure of the intestinal flora and regulating bile acid metabolism. The core physiological function of vitamin K is to act as a cofactor of γ-glutamyl carboxylase, catalyzing the carboxylation modification of specific glutamic acid residues, which is essential for the activation of coagulation factors (such as prothrombin) and some bone metabolic proteins. Vitamin K strengthens the intestinal barrier by activating PXR receptors, showing therapeutic potential in promoting liver detoxification, strengthening the intestinal barrier, and regulating pro-inflammatory and apoptotic pathways. In addition, vitamin K also has the function of preventing ferroptosis, which provides a new way to intervene in liver fibrosis. As a powerful antioxidant, vitamin C can directly scavenge ROS to reduce liver oxidative stress injury, thereby inhibiting HSCs activation and collagen expression. Its anti-fibrosis effect is also related to the regulation of TGF-β1 signaling pathway and inhibition of inflammatory cell infiltration. Unlike vitamins C and E, which directly neutralize ROS through electron transfer, B vitamins (especially folic acid and B12) do not act as direct antioxidants to eliminate free radicals. Instead, they mainly maintain the homeostasis of one-carbon unit metabolism, correct hyperhomocysteinemia (HHcy), ensure the supply of S-adenosylmethionine (SAMe)-dependent methyl donors and glutathione synthesis, thereby indirectly repairing the oxidative-reductive imbalance and inflammatory responses caused by metabolic disorders. Vitamin B6, as a key cofactor for the synthesis of GSH, when deficient, will exacerbate oxidative stress. Supplementing with B6 can help slow down the fibrotic process. Therefore, rational supplementation and combined application of B vitamins are of great significance for the prevention and treatment of liver fibrosis.

At the level of combined use of vitamins, there are complex synergistic or antagonistic effects between different vitamins. For example, the core of the synergistic antioxidant effect of vitamin E and vitamin C lies in the regenerative cycle mechanism. When vitamin E scavenges lipid peroxide free radicals (LOO•) in the lipid phase, it converts itself into α-tocopherol free radicals (TO). At this time, vitamin C in the aqueous phase reduces and regenerates TO• to active vitamin E through a single electron transfer reaction. At the same time, it converts itself into ascorbic acid free radicals, thereby restoring vitamin E's ability to continuously scavenge free radicals and significantly prolonging the inhibition time of antioxidant chain reaction (165–167). The synergistic effect of vitamin D and vitamin A nuclear receptors is mainly achieved by sharing the key dimerization partner retinol X receptor (RXR) (168, 169). The active metabolite of vitamin D, 1,25-dihydroxyvitamin D3 (1,25D), must form a VDR-RXR heterodimer with RXR after binding to vitamin D receptor (VDR), so as to effectively recognize and bind to VDREs in the promoter region of the target gene and initiate transcriptional regulation. At the same time, the active metabolite of VA, retinoic acid, plays a role by forming RAR-RXR heterodimer with RXR through RAR dependence (170). Vitamin A maintains the quiescent state and lipid droplet storage of HSCs through RAR-RXR (171). Vitamin D directly antagonizes the TGF-β/Smad signaling pathway through VDR-RXR. After activation of VDR, it can competitively inhibit the binding of Smad3 to genomic profibrotic elements, thereby blocking the core driving signal of HSCs activation (172, 173). At the same time, both of them can reduce liver inflammation load by regulating immune cell function. Vitamin B12 and folic acid can form a synergistic cycle in one-carbon metabolism. Folic acid takes THF as a carrier, carries one-carbon units of different oxidation states in the cytoplasm and mitochondria, and directly participates in the *de novo* synthesis of purine and thymidylate, amino acid metabolism and redox homeostasis maintenance (141). Vitamin B12, as an essential cofactor of methionine synthase (MTR/MetH), catalyzes the reaction of homocysteine with 5-methyltetrahydrofolate (5-methyl-THF) to generate methionine and release free THF simultaneously to support nucleotide synthesis, methylation reaction and redox homeostasis (174, 175). If B12 is deficient, folic acid will exist in 5-methyl-THF, leading to functional folic acid deficiency and interfering with DNA synthesis and epigenetic regulation (176). Abnormality of this pathway can lead to hyperhomocysteinemia (HHcy). Studies have shown that serum Hcy levels are positively correlated with liver inflammation and fibrosis. HHcy aggravates the progression of nonalcoholic steatohepatitis (NASH) to liver fibrosis through mechanisms such as Stx17 homocysteine. Vitamin B and folic acid supplementation can reverse liver injury in mouse models, improve liver histology and inhibit fibrosis-related gene expression (139). This provides a new idea for the optimization of future intervention strategies. However, combined vitamin supplementation strategies may also have antagonistic risks in clinical applications. For example, low serum retinol levels have been shown to be significantly associated with the degree of liver fibrosis and liver-related mortality (177), indicating that vitamin A status needs to be maintained within a precise window. As an endogenous agonist of PXR, vitamin K can regulate liver detoxification, intestinal barrier and inflammatory pathways, but it has a clear pharmacological antagonism with oral anticoagulants such as warfarin. It needs to be highly vigilant in the context of coagulation disorders in patients with cirrhosis (107). Therefore, instead of blindly combined supplementation, it is necessary to systematically analyze the vitamin-nuclear receptor-target organ axis. On the premise of clarifying the individual vitamin status, liver disease stage and drug background, the ratio, dose and timing should be scientifically designed, and a safe and effective intervention window should be delineated to achieve a balance between synergy and risk aversion (178).

However, the application of vitamins from nutritional supplements to precise therapeutic drugs for complex liver diseases still faces severe challenges in balancing safety and efficacy. The core difficulty is to determine the optimal therapeutic dose that can effectively activate the relevant therapeutic pathways and avoid toxic and side effects. Fat-soluble vitamins can accumulate in the body for a long time, and high-dose vitamin A supplementation will increase the risk of hepatotoxicity. Long-term high-dose intake of vitamin D may lead to hypercalcemia, which is more likely to occur in patients with chronic liver disease with calcium and phosphorus metabolism disorders. A cohort study of 3123 women of childbearing age found that participants with higher vitamin A and K levels were more likely to have irregular menstrual cycles (vitamin A: OR = 1.39 (95 % CI: 1.12,1.74); vitamin K: OR = 1.41 (95 % CI: 1.13, 1.76)) and longer menstrual cycle (vitamin A: OR = 1.34 (95 % CI: 1.06, 1.69); vitamin K: OR = 1.27 (95 % CI: 1.00,1.61)), and the relationship between the two showed a linear dose-response pattern, indicating that excessive serum levels may affect reproductive health (179). In addition, animal studies have shown that long-term intake of medium doses of vitamin D may also promote atherosclerosis (180). In patients with chronic kidney disease, routine supplementation of vitamin A and E is not recommended because of its potential toxicity risk (181). Although water-soluble vitamins are not easy to accumulate in the body and cause chronic poisoning, their application is also challenging. Intravenous injection of ultra-high doses of vitamin C may pose a risk of oxalate deposition. At the same time, due to its short half-life and fast excretion, frequent administration or continuous infusion is required to maintain effective blood concentration, which is difficult to operate in clinical practice. Although vitamins are safe as nutrients at conventional doses, their potential toxic and side effects must be strictly considered when pursuing super-physiological doses of therapeutic effects. Although this review systematically reviews the potential mechanisms of various vitamins in regulating inflammation and oxidative stress in liver fibrosis, it is necessary to recognize the limitations of current research. First of all, the existing evidence is mostly from basic research and animal experiments, and clinical research is not sufficient, especially the lack of well-designed randomized controlled trials, which limits the clinical application of vitamin anti-hepatic fibrosis effect. Secondly, most mechanism studies use unconventional doses of vitamin intervention, and whether it is sufficient to trigger the above pathways at the level of dietary intake remains to be verified. Future research should conduct stratified intervention trials based on vitamin-deficient populations to clarify the characteristics of the beneficiary groups, explore appropriate supplementation doses and treatment courses, seek a balance between effectiveness and safety, pay attention to the synergistic effects of combined vitamin use and their interactions with existing antifibrotic drugs, focus on in-depth exploration of mechanisms, explore the application of vitamins in regulating autophagy, mitochondrial function, epigenetic modifications and other aspects in liver protection, and combine emerging technologies to identify biomarkers that predict vitamin responsiveness, achieving targeted and precise intervention.

This review systematically explores the potential mechanisms by which various vitamins regulate inflammation and oxidative stress in basic research. In clinical settings, patients with chronic liver diseases often fall into a state of vitamin deficiency due to multiple factors, resulting in reduced vitamin intake, absorption disorders, decreased reserves, and increased consumption. For healthy individuals with good nutritional status, additional supplementation of vitamins is unlikely to bring any additional anti-fibrotic benefits, and may even increase the risk of toxicity due to the accumulation of fat-soluble vitamins. Therefore, vitamin intervention should be precisely targeted at the subgroups of patients with liver fibrosis who have a clearly identified risk of deficiency. For patients with liver fibrosis who can consume orally, dietary supplementation should be the first choice. Increasing the intake of natural foods rich in specific vitamins can not only avoid excessive risks but also benefit from other synergistic components in the food. When dietary adjustments are insufficient to correct the deficiency, low-dose supplements can be considered to reach the lower or median limit of the normal physiological range, rather than aiming for supra-physiological levels. For patients with severe absorption disorders, such as those with cholestatic liver cirrhosis, water-soluble vitamins or short-term moderate-dose supplementation can be administered under the supervision of medical staff. The mechanism of action of vitamins discussed in this article is mainly based on the maintenance of the basic antioxidant defense system and immune homeostasis after restoring the vitamin levels of patients with liver fibrosis to the normal physiological range. Future clinical research should focus on establishing diagnostic criteria for vitamin deficiency in patients with liver fibrosis, conducting stratified intervention trials based on the deficient population, and exploring appropriate supplementation doses and treatment courses to achieve a balance between efficacy and safety. In summary, the clinical value of vitamins in the prevention and treatment of liver fibrosis lies mainly in their potential to provide precise alternative treatment for specific deficient populations, and it points out the direction for subsequent research.

In summary, vitamins, with their unique antioxidant, anti-inflammatory and signal regulatory capabilities, have shown broad prospects in the prevention and treatment of liver fibrosis. However, in the process of their clinical application, a profound understanding of metabolic changes under disease conditions, individual differences, complex mechanism networks and potential toxic risks is a prerequisite for safe and effective application. This article systematically reviews the core advantages and mechanisms of action of vitamins as natural antioxidants in anti-inflammation and anti-oxidative stress in liver fibrosis from the molecular mechanism level. The aim is to provide a reference basis for the subsequent clinical application of vitamins as anti-inflammatory and antioxidant agents in the treatment of liver diseases.
